# Biodefense Implications of New-World Hantaviruses

**DOI:** 10.3389/fbioe.2020.00925

**Published:** 2020-08-07

**Authors:** Michael Hilary D’Souza, Trushar R. Patel

**Affiliations:** ^1^Department of Chemistry and Biochemistry, Alberta RNA Research and Training Institute, University of Lethbridge, Lethbridge, AB, Canada; ^2^Department of Microbiology, Immunology and Infectious Disease, Cumming School of Medicine, University of Calgary, Calgary, AB, Canada; ^3^Li Ka Shing Institute of Virology and Discovery Lab, University of Alberta, Edmonton, AB, Canada

**Keywords:** hantavirus, Sin Nombre Virus, Andes Virus, biodefense, bioterrorism, viral pandemic, hantavirus cardiopulmonary syndrome, transmission

## Abstract

Hantaviruses, part of the *Bunyaviridae* family, are a genus of negative-sense, single-stranded RNA viruses that cause two major diseases: New-World Hantavirus Cardiopulmonary Syndrome and Old-World Hemorrhagic Fever with Renal Syndrome. Hantaviruses generally are found worldwide with each disease corresponding to their respective hemispheres. New-World Hantaviruses spread by specific rodent-host reservoirs and are categorized as emerging viruses that pose a threat to global health and security due to their high mortality rate and ease of transmission. Incidentally, reports of Hantavirus categorization as a bioweapon are often contradicted as both US National Institute of Allergy and Infectious Diseases and the Centers for Disease Control and Prevention refer to them as Category A and C bioagents respectively, each retaining qualitative levels of importance and severity. Concerns of Hantavirus being engineered into a novel bioagent has been thwarted by Hantaviruses being difficult to culture, isolate, and purify limiting its ability to be weaponized. However, the natural properties of Hantaviruses pose a threat that can be exploited by conventional and unconventional forces. This review seeks to clarify the categorization of Hantaviruses as a bioweapon, whilst defining the practicality of employing New-World Hantaviruses and their effect on armies, infrastructure, and civilian targets.

## Introduction

**FIGURE 1:**
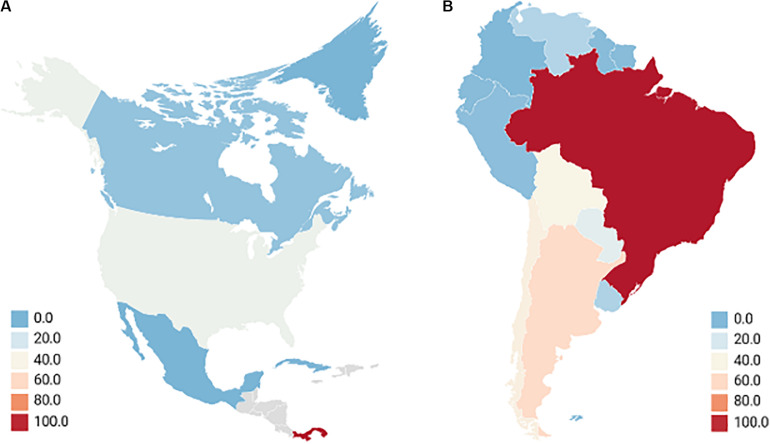
Annual average cases of new-world hantaviruses in the western hemisphere. North America **(A)** although Mexico has reported there being no HCPS cases, seroprevalence of hantaviruses exists in 10.15% of rodents, much of which occurs in Mexican states that border the United States where 299 cases of HCPS were reported between 1993 and 2017. Seropositive humans were identified, and the lack of reporting is attributed to the febrile disease being misconstrued with other illnesses (Vigueras-Galvan et al., 2019). This is very similar to other Central Latin American nations which have very limited reporting or insufficient data but show seroprevalence of hantavirus in rodents, up to 20.8% in Honduras as an example (Knust and Rollin, 2013; Rovida et al., 2013; Montoya-Ruiz et al., 2014; Drebot et al., 2015). Gray locations indicate countries with no reporting of hantavirus. South America **(B)** most cases occurred in rural or forested environments with farming being a major concern. Reporting is an issue as the actual annual cases for Brazil, Columbia, and Venezuela are considered to be significantly higher (Figueiredo et al., 2014; Montoya-Ruiz et al., 2014; Goeijenbier et al., 2015; Riquelme et al., 2015; Jiang et al., 2017; Matheus et al., 2017; Fonseca et al., 2018; Alonso et al., 2019; Escalera-Antezana et al., 2020). Averages of HCPS-causing hantavirus cases taken from studies carried out between 2000 and 2019.

Hantaviruses are emerging zoonotic viruses that are responsible for two human diseases: Hantavirus Cardiopulmonary Syndrome (HCPS) associated with New-World Hantaviruses found in the western hemisphere; and Hemorrhagic Fever with Renal Syndrome (HFRS) associated with Old-World Hantaviruses in the eastern hemisphere (Mittler et al., 2019). Collectively, 150,000 – 200,000 cases of hantavirus disease are reported annually with the majority of HFRS cases occurring in Asia, specifically in the People’s Republic of China which constitutes upwards of 90% of cases (Schmaljohn, 2009; Iannetta et al., 2019; Liu et al., 2019). HCPS, comparatively, presents a stark minority in annual cases, roughly 300, with the majority of cases being in South America and primarily Brazil (Watson et al., 2014; Duehr et al., 2020). The annual average cases of New-World HCPS-causing hantaviruses in the western hemisphere are summarized in Figure 1. Both HFRS and HCPS exhibit drastically different mortality rates, with the former causing upwards of 12% while the latter inflicting upwards of 35–50% mortality on infected persons (Jonsson et al., 2010; Llah et al., 2018). Due to hantaviruses being emerging pathogens with HCPS-causing infections retaining a high mortality rate, there remains a possible risk of hantaviruses being engineered into novel bioweapons (Meyer and Morse, 2008; Kruger et al., 2011; Williams and Sizemore, 2020).

Biological systems that can potentially be used as weapons have been divided into three groups designated Category A, B, and C (Table 1) (Christian, 2013). Category A agents are described as organisms or toxins that pose a national security risk because they can be easily transmitted or disseminated, can result in high mortality with a major public health impact, can cause public panic and social disruption, and require special action to ensure public health preparedness (Christian, 2013). Category A agents include *Bacillus anthracis*, *Clostridium botulism* neurotoxin, and viral hemorrhagic fever viruses such as Ebola and Marburg. Category A agents are especially important because of their high mortality rate and rapid disease progression. For example, the spores of *B. anthracis* are highly resistant to adverse environmental conditions such as heat, cold, humidity, and radiation (Doganay and Demiraslan, 2015). These spores are easily produced in laboratories, dried, and refined as a powder that can be released as an aerosol, which if inhaled, can result in inhalation anthrax, meningitis, and bacteremia. If untreated, the disease is highly fatal (Miroslav, 2020).

**TABLE 1:** Centers for Disease Control and Prevention (CDC) Biological Agent Categories (Christian, 2013; CDC, 2018).

**Category**	**Category definition**	**Diseases**	**Organisms and agents**
A	High-priority agents that include organisms that pose a risk to national security because they: • Can be easily disseminated or transmitted person-to-person • Result in high mortality and have the potential to cause a major public health impact • Might cause public panic and social disruption • Require special action for public health preparedness	Anthrax Botulism Plague Smallpox Tularemia Viral Hemorrhagic Fevers (VHFs)	Bacillus anthracis Clostridium botulinum toxin Yersinia pestis Variola major Francisella tularensis Filoviruses (Ebola, Marburg) Arenaviruses (Lassa, Machupo)
B	Second highest priority agents which include those that: • Are moderately easy to disseminate • Result in moderate morbidity rates and low mortality • Require specific enhancements of laboratory capacity and enhanced disease surveillance	Brucellosis Epsilon Toxin Food Safety Threats Glanders Melioidosis Psittacosis Q Fever Ricin Toxin Staphylococcal Enterotoxin B Typhus Fever Viral Encephalitis Water Safety Threats	Brucella species Clostridium perfringens Salmonella species Escherichia coli O157:H7 Shigella Burkholderia mallei Burkholderia pseudomallei Chlamydia psittaci Coxiella burnetii Ricinus communis (Castor beans) Staphylococcus aureus Rickettsia prowazekii Alphaviruses (Venezuelan Equine Encephalitis, Eastern Equine Encephalitis, Western Equine Encephalitis) Vibrio cholerae Cryptosporidium parvum
C	Third highest priority agents include emerging pathogens that could be engineered for mass dissemination in the future because of: • Availability • Ease of production and dissemination • Potential for high morbidity and mortality causing major health impacts	Emerging Infectious Diseases	Nipah Virus Hantaviruses Tick-Borne Hemorrhagic Fever Viruses Tick-Borne Encephalitis Viruses Yellow Fever Multidrug-Resistant Tuberculosis

Category B agents are the second-highest priority agents and typically include agents that are responsible for moderate morbidity and low mortality rates, and are moderately dispersible (Christian, 2013). They tend to include food safety threats and diseases from toxins like the Ricin toxin from Castor beans (*Ricinus communis*) that can be employed in local attacks and assassinations that have a low death rate compared to Category A agents but can still inflict significant damage to political and social systems (Bhalla and Warheit, 2004). This contrasts with the third-highest priority, Category C Pathogens which can include emerging pathogens that could be engineered for mass dissemination through their: availability, ease of production and dissemination; and their potential for high mortality resulting in a major health impact (Christian, 2013). Hantavirus weaponization is speculative as there are no known major weapon development programs occurring. However, their weaponization remains attractive due to their potential to cause high mortality (up to 60% during the height of the 1993 Four Corners outbreak), and their ability to target young and healthy adults in risk occupations such as agriculture and forestry (Hjelle et al., 1994; de St Maurice et al., 2017). Ultimately, the priority difference between the two doesn’t make one any less meaningful, as the employment of bioagents from any category of bioweapon could have a public health impact with implications to national security.

Category considerations of biological agents vary between the Centers of Disease Control and Prevention (CDC) and the US National Institute of Allergy and Infectious Diseases (NIAID) based upon circumstance of the infectious agent. The CDC assesses bioagent risks in support of US public health systems and primary healthcare providers and how, based upon the categorization, they should respond to biological agents and pathogens including those that seldom occur in the US (CDC, 2018). NIAID’s categories refer to documented priority pathogens A, B, and C, and emerging infectious diseases defined as those that have newly appeared in a population or have existed but are rapidly increasing in incidence or geographic range (NIAID, 2018). There is much categorical confusion though for hantaviruses, and specifically New-World Hantaviruses such as Sin Nombre *orthohantavirus* (SNV), as to what priority of a bioagent and subsequent threat they pose (Mir, 2010; Mittler et al., 2019). Hantaviruses as a whole are categorized as emerging viruses along with Nipah Virus in the CDC as Category C Pathogens; whereas NIAID places hantaviruses as part of the Category A Pathogens (CDC, 2018; NIAID, 2018). Hantaviruses, specifically Old-World Hantaviruses causing HFRS, are listed in Category C due to their shared symptoms to other agents causing Viral Hemorrhagic Fever (VHF) that cause capillary leakage syndrome and hemorrhaging (Clement, 2003). Category C retains the lowest priority of risk to national security and ultimately the lowest potential as a biowarfare agent. However, it doesn’t diminish the risk that hantaviruses pose globally. With their widespread nature, being present on every continent except for Australia and Antarctica, hantaviruses continue to pose a risk to human systems and activities that closely engage with their rodent-specific reservoirs including military personnel, agricultural workers, and transport industries including warehouse and shipping staff (Forbes et al., 2018). This review paper seeks to clarify the categorization of hantaviruses as bioweapons as well as to define the practicality of employing hantaviruses, specifically HCPS-causing SNV and Andes Virus (ANDV), as novel bioagents against modern militaries and industries.

## HCPS-Causing New-World Hantaviruses

**FIGURE 2:**
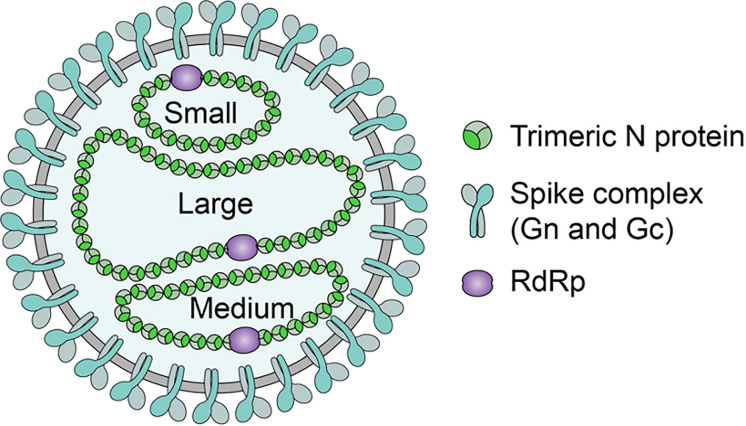
Hantavirus structure. Hantaviruses are enveloped with a lipid bilayer containing Glycoprotein spikes assemblies comprised of Gn and Gc Glycoproteins. Contained within the envelope are the equimolar amounts of N Protein packaged S (small), M (medium), and L (large) segments vRNA, which are associated with an RdRp (Hussein et al., 2011; Hepojoki et al., 2012). SNV structure is generally spherical with a dense envelope (Goldsmith et al., 1995). N Protein forms trimers that selectively encapsidates the negative-sense vRNA into RNPs and assists in its panhandle formation for packaging (Mir and Panganiban, 2004).

The hantavirus genus forms part of the *Bunyavirus* family and is composed of well-defined serotypes that are each associated with a specific primary rodent reservoir (Spiropoulou et al., 1994; Avsic-Zupanc et al., 2019). The hantavirus genome is tripartite and is composed of three segments of negative-sense, single-stranded RNA (Nichol et al., 1993). The three segments are organized by size and are designated as the Large (L), Medium (M), and Small (S) segments since the tripartite genome lengths are generally 6.6, 3.7, and 2.1 kb for the L, M, and S segments respectively (Figure 2) (Plyusnin et al., 1996). The genomic L, M, and S segments encode for the 250 kDa RNA-dependent RNA polymerase (RdRp), 125–127 kDa Glycoprotein Precursor (GPC) and subsequent co-translationally cleaved Gn and Gc Glycoproteins, and the 48 kDa Nucleocapsid (N) Protein respectively (Nichol et al., 1993; Kamrud and Schmaljohn, 1994; Chizhikov et al., 1995). SNV, amongst other hantaviruses with the exception of Haantan Virus (HTNV), Seoul Virus (SEOV), and Dobrava Virus (DOBV), have an open reading frame (ORF) for a putative Non-Structural Protein (NSs ranging between 7 and 10 kDa in size) (Plyusnin et al., 1996). Additionally, each genomic segment is flanked by 5′ and 3′ Non-Coding Terminal Regions (NTRs) which are common to Bunyaviruses (Amroun et al., 2017).

**FIGURE 3:**
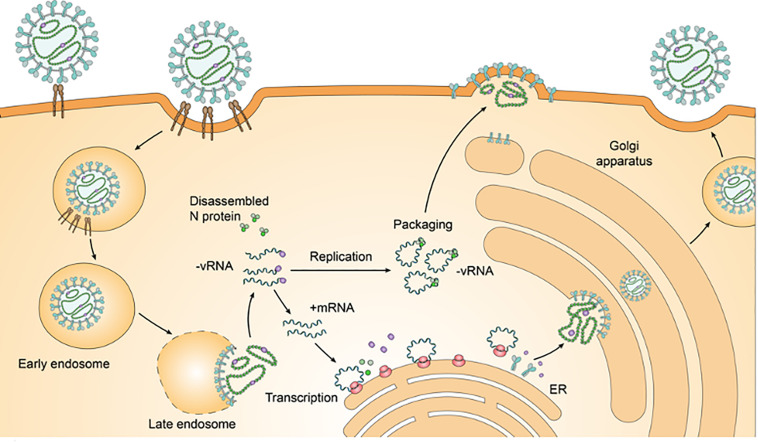
HCPS-causing hantavirus life cycle. Pathogenic HCPS-causing hantaviruses such as SNV or ANDV will first bind to β_3_-Integrin receptors on endothelial cells which will mediate endocytosis. The formation of an early endosome helps traffic the virion to the Golgi Complex. Following a pH-mediated membrane fusion, the now late endosome disassembles and releases the ribonucleoproteins (RNPs) near the Endoplasmic Reticulum-Golgi Intermediate Compartment (ERGIC). The RNPs disassemble and the RdRp carries out transcription and replication in the cytoplasm, cleaving cellular mRNA to form capped primers to initiate viral mRNA transcription. Transcribed S, M, and L Segment mRNA is translated into N Protein, GPC (and then into co-translated Gn and Gc Glycoproteins), and RdRp respectively. Negative-sense vRNA serves as the template for the transcription of mRNA. RdRp undergoes a transition from transcription to the replication of negative-sense vRNA which is considered to be mediated by the increase in free N Protein concentration (Jonsson and Schmaljohn, 2001). New-World Hantaviruses will be assembled at the plasma membrane compared to Old-World Hantaviruses that are assembled at the Golgi complex (Muyangwa et al., 2015). Nascent virions bud from the plasma membrane.

The L Segment’s RdRp acts as the RNA transcriptase and replicase, transcribing mRNA and replicating the genomic RNA using the positive-sense RNA as an intermediate (Kukkonen et al., 2005) (Figure 3). Hantaviral RNA segments are each associated with the RdRp and are packaged within a ribonucleoprotein complex formed by the N Protein (Hepojoki et al., 2012). The RdRp is responsible for vRNA transcription and replication, additionally retaining endonuclease activity which is used to cleave the 5′-termini of host mRNA to act as a primer which initiates viral mRNA transcription in a process called cap-snatching and prime and realignment (Kukkonen et al., 2005). This occurs in conjunction with the N Protein which is found to form an N-RdRp complex for RNA synthesis whilst also binding to mRNA caps by recognizing a five nucleotide sequence adjacent to the 5′ cap for high-affinity binding (Mir et al., 2010; Cheng et al., 2014). During cap-snatching, the viral RdRp binds to methylated capped 5′ ends of host mRNAs and cleaves them for use as a primer for mRNA synthesis with a preference for host mRNAs that contain a Guanine prior to the cleave site (Garcin et al., 1995). The prime and realignment follow the methylated 5′ cap whose aforementioned G nucleotide at the −1 position would align opposite a Cytosine nucleotide at the +3 position on the negative-sense vRNA genome. After the primer is extended up to 3 nucleotides, the nascent chain will realign to shift the original 3′ Guanine back to the −1 position ultimately generating two to four UAG repeats (Hutchinson et al., 1996).

Pathogenic hantaviruses retain glycoproteins that target and interact with the β_3_ chain of Integrins that is especially abundant as a surface receptor on endothelial cells, dendritic cells (DC), and platelets where they are critical in maintaining capillary integrity (Gavrilovskaya et al., 1998). Endothelial cells are ubiquitously infected throughout the body by HFRS and HCPS-causing hantaviruses, however, pulmonary endothelial cells are the primary targets during HCPS infections with DCs and platelets being involved in the pathogenic process of vascular leakage and thrombocytopenia (Connolly-Andersen et al., 2014, 2015; Ermonval et al., 2016). Hantaviruses inducing HCPS employ α11β3 Integrins with hantaviruses inducing HFRS employing ανβ3 Integrins for entry both of which are β_3_ Integrins; α5β1 Integrins are employed by non-pathogenic hantaviruses (Gavrilovskaya et al., 1998, 1999). The hantavirus virion is itself enveloped retaining a lipid bilayer whose membrane is 5 nm thick and studded with the Gn and Gc glycoprotein spike assemblies that project 10 nm from the membrane in fourfold rotational symmetry (Hepojoki et al., 2012). The virion’s shape appears as a rounded, pleiomorphic particle ranging between 70 and 350 nm in diameter (Plyusnin et al., 1996; Hepojoki et al., 2012). The M Segment retains a five amino acid sequence (WAASA) that precedes the co-translational cleavage site for the GPC which is conserved across all hantaviruses (Spiropoulou et al., 1994).

The N Protein’s role is multifaceted, but is primarily involved in the encapsidation of the vRNA and protects it from host cellular nucleases by binding selectively to Hantaviral panhandle structures (Mir and Panganiban, 2005**;** Mir et al., 2010). Each SNV segment possesses conserved terminal sequences at the 5′ and 3′ NTRs that are capable of complementarily base-pairing to form panhandle structures (Chizhikov et al., 1995). These conserved sequences, consisting of 14–17 nucleotides, were found to be highly conserved throughout the hantavirus genus, being comprised of the following sequence at the 3′-termini: 3′AUCAUCAUCUGAGG-5′; and the following sequence at the 5′-termini: 5′-UAGUAGUAU(G/A)CUCC-3′ (Chizhikov et al., 1995). Trimeric N Protein subsequently recognizes these panhandle structures with specificity and encapsidates the vRNA, with trimerization being required for high-affinity binding (Mir and Panganiban, 2004**;** Brown and Panganiban, 2010). The N Protein is also genus-specific and can bind to vRNA and cRNA of other hantavirus species (Mir et al., 2006). The N Protein’s RNA chaperone roles are employed when interacting with the vRNA and its panhandle structures, assisting in the dissociation of the RNA duplexes and initiating replication by the RdRp (Kukkonen et al., 2005**;** Mir and Panganiban, 2006a). The N Protein’s RNA chaperone properties also enable it to bind to misfolded vRNA, refolding it to allow the high-ordering of panhandle structures to form and to prevent RNA structures from falling into inoperable kinetic traps (Mir and Panganiban, 2006b).

SNV and ANDV are the two major causative agents of HCPS, with ANDV occurring in South America which is spread by the *Sigmondontinae* subfamily and mainly by *Oligoryzomys longicaudatus* or the long-tailed pygmy rice rat reservoir (Beltran-Ortiz et al., 2017; Astorga et al., 2018). Before the presence of SNV, hantaviruses were not considered to be a serious public health threat as other hantaviruses like Prospect Hill Virus (PHV) and HFRS-causing SEOV have been found in a number of US cities (Spiropoulou et al., 1994). The disease took significant attention when the respiratory illness of HCPS was first observed in the Four Corners region of the United States in 1993, with the outbreak causing upwards of 75% mortality in healthy adults between the ages of 20–40 years (Nichol et al., 1993). The deer mouse (*Peromyscus maniculatus*) was identified as the primary rodent reservoir for SNV; it is one of the most abundant small mammals in North America, found not just exclusively to the Canadian prairies and the American Midwest (Spiropoulou et al., 1994). The emergence of SNV is likely due to environmental factors that favored the natural reservoir of deer mice to increase, allowing for increasing opportunities for human infection (Schmaljohn and Hjelle, 1997). The reoccurrence and increased rodent-human contact can be attributed to increased food availability from erratic weather conditions that produced higher precipitation and warmer climates (Watson et al., 2014). Due to climate change and its impact of lowering biodiversity caused a dilution effect that altered reservoir behavior and forced population migration, and the ultimate spread of the infectious agents to human systems (Watson et al., 2014).

The reservoirs themselves can spread the virus horizontally, being nearly asymptomatic but chronically infected (Guterres and de Lemos, 2018). HFRS and HCPS are generally acquired from the inhalation of aerosolized excreta including feces, urine, and saliva infected with hantavirus (Kariwa et al., 1998**;** Kallio et al., 2006**;** Godoy et al., 2009**;** Warner et al., 2019a). This can also include the direct contamination of food or household articles with rodent excreta as well as virion particles shed from rodent skin and fur (Yanagihara et al., 1985**;** Zaki et al., 1995). However, SNV transmission requires direct contact between SNV-infected rodents and humans as contaminated cages proved to be ineffectual in transmitting the disease horizontally to uninfected deer mice (Warner et al., 2019a). ANDV is unique in that it can transmit hantavirus through person-to-person contact (Alonso et al., 2020). No other hantavirus exhibits the property of person-to-person transmission, which makes ANDV a preferable candidate for weaponization, which would take advantage of the additional spread mechanism. No person-to-person hantavirus infections have been reported in North America, making SNV less effective as a bioweapon, comparably (Hartline et al., 2013). The person-to-person transmission of ANDV occurs mainly in family clusters or close activities with infected persons during the disease’s prodrome phase, occurring during the interval of 12–27 days between the initial exposure and the onset of symptoms (Figueiredo et al., 2014). Sexual partners have a higher risk of infection compared to non-sexual partners (Martinez-Valdebenito et al., 2014).

Hantaviruses generally enter cells utilizing a clathrin-dependent pathway which follows the formation of an early endosome and subsequent low-pH initiating dissolution of the late endosome for infectious entry (Jin et al., 2002) (Figure 3). SNV and ANDV can enter endothelial cells primarily by a receptor-mediated endocytic pathway involving β_3_-integrins but also a clathrin-dependent pathway (Gavrilovskaya et al., 1998; Chiang et al., 2016). New-World Hantaviral replication occurs predominately in pulmonary endothelial cells which have exhibited the highest viral loads, resulting in increased vascular permeability (Zaki et al., 1995). Macrophages, follicular DCs, and DCs are also known to replicate the Hantaviral genome with the virus also being found in human tissues of the kidney, spleen, pancreas, lymph nodes, skeletal muscles, heart, intestines, adipose tissue, urinary bladder, and brain (Borges et al., 2006). Replication occurs in the cytoplasm with the budding of the Hantaviral virion occurring in the Endoplasmic Reticulum Golgi Intermediate Complex (ERGIC); SNV uniquely, but principally, buds from the plasma membrane (Goldsmith et al., 1995; Vapalahti et al., 2003) (see Figure 3).

Hantavirus infections activates the innate immune system with downstream effects that induces disease. The innate immune system recognizes pathogens through their interaction with Pattern Recognition Receptors (PRRs) which are expressed by many cell types, including endothelial and epithelial cells. Viruses present Pathogen-Associated Molecular Patterns (PAMPs) which are recognized by PRRs which activate signaling cascades and transcription factors that modulate the expression of type I Interferons and Interferon-Stimulating Genes (ISGs) involved in antiviral functions. Toll-like Receptors (TLRs) and Retinoic acid-Inducible Gene-I (RIG-I), including the RIG-I-like receptor Melanoma Associated Gene 5 (MDA5), are PRRs that are involved in the recognition of pathogenic RNA viruses by binding to vRNA (Kawai and Akira, 2007). Binding of PAMPs in the form of vRNA to TLRs and RIG-I receptors activates transcription factors NF-κB and IFN Regulatory Factor 3 and 7 (IRF3/7) that are translocated into the nucleus to bind to ISGs that are used to express Interferons (IFN) (Sen and Sarkar, 2005; Kato et al., 2006). Type I Interferons (IFN-α/β) are critical regulators of immune cell activation, development toward antiviral activity, cell growth, and apoptosis and are involved in stimulating the Janus Kinases and signal and activators of transcription pathways (JAK/STAT) (McNab et al., 2015).

New World Hantaviral proteins antagonize virus recognition by suppressing the JAK/STAT signaling pathways with ANDV utilizing its N Protein and the GPC to disrupt antiviral activity while SNV employs its GPC alone (Levine et al., 2010). The N Protein of hantaviruses has been reported to inhibit IFN activity and NF-κB activation, with ANDV N Protein inhibiting signaling responses instigated by RIG-I and MDA5 and upstream IRF3 phosphorylation (Taylor et al., 2009; Cimica et al., 2014; Pan et al., 2015). Both HTNV and ANDV N Proteins inhibit Tumor Necrosis Factor α induced activation from NF-κB by preventing the transcription factors translocation into the nucleus (Taylor et al., 2009). Additionally, reports identified the highly conserved domains of the Glycoprotein Gn’s cytoplasmic tail which also functions in early IFN responses by blocking IRF3 and NF-κB activation and subsequent downstream antiviral function of the early immune response (Matthys and Mackow, 2012; Mackow et al., 2014). Vero cell lines are used preferably to isolate and amplify hantaviruses since they are deficient in IFN-I and IFN-II expression and will not elicit an immune response to infection although New-World Hantaviruses have been shown to elicit IFN-λ activation in Vero cell lines (Emeny and Morgan, 1979; Prescott et al., 2010; Seto et al., 2011). Pre-treatment of IFN-λs have been shown to induce antiviral activity against HTNV infection by activating the JAK-STAT pathway in A549 cells (Li et al., 2019). Pathogenic hantaviruses tend to regulate the early induction of IFN to replicate successfully with pretreated Type I IFNs only being successful shortly after infection (Matthys and Mackow, 2012).

## Symptoms

Patients suffering from HCPS generally present fever, headache, muscle aches, and chills as well as leukocytosis and thrombocytopenia, which rapidly progresses to more severe respiratory diseases (Schmaljohn and Hjelle, 1997). After 4–10 days, individuals infected with HCPS-causing hantaviruses developed influenza-like illnesses followed by rapidly progressing pulmonary edema caused by pulmonary capillary leak syndrome, resulting in respiratory dysfunction and shock (Zaki et al., 1995; Schmaljohn, 2009). HCPS is particularly important because unlike other respiratory diseases, it occurs in young, healthy adults (Beltran-Ortiz et al., 2017). Death occurred 2–10 days after the onset of the illness within almost 50% of patients observed (Borges et al., 2006). Although HCPS shares some similarities with HFRS, like the febrile prodrome and capillary leakage, the kidneys are largely unaffected with capillary leakages occurring exclusively in the lungs and resulting in shock and cardiac complications despite sufficient tissue oxygenation (Schmaljohn and Hjelle, 1997).

## Vaccines and Therapeutics

There are no US FDA-approved vaccines available for hantavirus infections, however, there are a variety of live-attenuated vaccines (Hantavax), DNA vaccines, subunit vaccines, and virus-like particle (VLP) vaccines that all demonstrate varying degrees of effectiveness (Schmaljohn et al., 1992; Cho and Howard, 1999; Choi et al., 2003; Ying et al., 2016; Liu et al., 2019). The Hantavax vaccine is available and is instituted in the Republic of Korea, with effectiveness against HFRS-causing hantaviruses such as HTNV and SEOV and resulting in a subsequent reduction in HFRS-related hospitalizations (Yi et al., 2018). However, its immunogenicity is dependent on early booster vaccinations in tandem with its two-dose primary vaccination which was demonstrated to provide timely protection to high-risk groups like farmers and those in the military (Song et al., 2016). DNA vaccines that use recombinant Vesicular-Stomatitis virus vectors expressing SNV and ANDV glycoproteins in Syrian hamster models were also effective at eliciting an immune response and conferred protection against lethal ANDV (Warner et al., 2019b). DNA vaccines are preferable because they can present the most immunogenic antigens to the host immune system whilst avoiding the need to propagate inactivated hantaviruses that are universally difficult to grow, isolate, and purify, with many DNA vaccines expressing Old-World Hantavirus glycoprotein genes and eliciting successful immune responses in hamster models (Schmaljohn et al., 2014).

There are currently no US FDA-approved post-exposure therapeutics against Hantaviral infections, however, there are treatment strategies present to manage HFRS and HCPS (Liu et al., 2019). Virus-targeting antivirals including antiviral drugs, antibodies, or novel-small molecules are designed to block hantavirus entry or to reduce viral replication. Ribavirin is an effective anti-Hantaviral drug that affects the biological function of RdRp and has had some success in treating HFRS cases including protecting Syrian hamsters in lethal HCPS models (Safronetz et al., 2011; Warner et al., 2019b). Ribavirin was effective at preventing lethal HCPS disease by having an inhibitory effect on ANDV replication (Safronetz et al., 2011). Ribavirin also inhibits SNV *in vitro* while the pre-treatment of deer mice followed by daily therapy of Ribavirin reduced SNV infection and viral RNA synthesis (Medina et al., 2007). However, Ribavirin has some limitations as at high doses it is toxic to humans and animals and causes anemia (McKeeJr., Huggins et al., 1988; Chapman et al., 1999). It was also noted that intravenous Ribavirin was ineffective at treating HCPS-patients after the onset of the cardiopulmonary phase (Chapman et al., 1999; Mertz et al., 2004). Antivirals also function effectively only during the early infection stage and not after the start of viremia (Brocato and Hooper, 2019). This could largely be attributed to the uncontrolled immune response which predominates the Hantaviral pathogenesis process after immediate infection (Liu et al., 2019). The Hantaviral prodrome phase can also be difficult to differentiate from other febrile illnesses, which may benefit infection by impeding proper identification and treatment (Brocato and Hooper, 2019). Another antiviral is Favipiravir that has shown broad-spectrum antiviral activity against RNA viruses including Bunyaviruses, being better than Ribavirin in that it is well-tolerated in humans without hemolytic anemia related side effects (Safronetz et al., 2013). Favipiravir was evaluated using *in vivo* studies for both SNV and ANDV infected hamster lethal disease models and resulted in complete survival as well as the reduction of ANDV RNA and antigens in the blood and lungs, although it was no longer effective after the onset of viremia in delayed antiviral treatment studies (Safronetz et al., 2013; Brocato and Hooper, 2019).

Hantaviruses can be inactivated by heat (sustained 30 min at 60°C), detergents, UV radiation, organic solvents, and hypochlorite solutions (Avsic-Zupanc et al., 2019). Despite this, hantaviruses are fairly durable and unexpectedly stable outside of a host, being able to survive longer than 10 days at room temperature and more than 18 days between the −20 and 4°C range (Vaheri et al., 2013). For most hantaviruses, contaminated dust or aerosols can transmit the virus to other rodents for up to 15 days after being excreted with viral infectivity in the culture being lost within 5–11 days when incubated at 23°C (Kallio et al., 2006; Hardestam et al., 2007). 70% Ethanol completely inactivates Bunyaviruses broadly, with HTNV being partially resistant to 30% Ethanol (Hardestam et al., 2007). These are largely chemical prophylactics designed to maintain sanitation and treat hantavirus-contaminated facilities and would create risk for livestock and personnel unprotected by strong detergents or hypochlorite solutions. Consequently, the absence of any effective vaccines or therapeutics makes hantavirus infections particularly dangerous to those working or operating in risk environments including agriculture, forestry, mining, and military operations.

## Biowarfare Potential of HCPS-Causing Hantaviruses

Hantaviruses have generally remained in the Category C position from the CDC and biodefense categorizations which is different from the laboratory biosafety criteria summarized in Table 2. Hantaviruses are considered a biosafety level 3 bioagent with regards to NIH and across the European Union (EU), with the exception of HCPS-causing hantaviruses in the EU being considered a level 2 because their criteria differ with regards to an agent that causes human disease and might be a hazard to workers, but is unlikely to spread to the community, and there is usually effective prophylactic treatment available (Tian and Zheng, 2014). For comparison, the NIH treats Ebola virus as a level 4; *Bacillus anthracis* as a level 2; SARS-associated coronavirus (SARS-CoV) as a level 3; and Human Immunodeficiency Virus (HIV) as a level 3; regardless of the biosafety levels, both Ebola Virus and Anthrax are considered very high biothreats with hantavirus being a high threat in the EU (Tian and Zheng, 2014). BSL 3 laboratory requirements are intensive, especially for highly pathogenic diseases that can cause harm to materials and personnel. Incidentally, for a research or industrial laboratory to study hantaviruses they require: direct physical protection from the virus in the form of PPE including gloves, masks, gowns, respiratory protection, and positive pressure ventilation suits; Biosafety Cabinets (BSC) as primary containments to isolate the pathogen and the user; secondary containments to mitigate or prevent the pathogen’s presence outside the BSC and its exit outside BSL 3 containment; and physical barriers in the form of walls, fences, or exclusion zones to prevent outside contamination (Pastorino et al., 2017). The initial infrastructural costs and maintenance of BSL 3 containment protocols would be prohibitively expensive and complex for uninitiated bioterrorist organizations making its development by smaller, resource poor organizations unfeasible.

**TABLE 2:** Biosafety categorization based upon the National Institute of Health (NIH) Criteria (Tian and Zheng, 2014).

	**Requirements**
1	Agents that are not associated with disease in healthy adult humans.
2	Agents that are associated with human disease which is rarely serious and for which preventive or therapeutic interventions are often available.
3	Agents that are associated with serious or lethal human disease for which preventive or therapeutic interventions may be available (can cause high individual risk but low community risk).
4	Agents that are likely to cause serious or lethal human disease for which preventive or therapeutic interventions are not usually available (can cause high individual risk and high community risk).

Hantaviruses are cited as being possible bioweapons that can be used against humans. When focused on specific serotypes of hantavirus, like SNV and ANDV, it becomes apparent that with their high mortality rate and rapid disease course with serious cardiopulmonary symptoms New-World Hantaviruses as opposed to HFRS-causing Old-World Hantaviruses are the more severe threat (Jonsson et al., 2008). Since HCPS-causing New-World Hantaviruses exhibit a high mortality (up to 50% in older patients) but low morbidity, it would preclude them from the Category B bioweapons which specifically are classified by their moderate morbidity and low mortality rates (Drebot et al., 2015). This causes SNV or ANDV bioagents to be assessed within Category A or C terms, although the moderate dissemination quality of Category B is reflective of hantaviruses and their limited projection by aerosols and rodents. Nevertheless, successful bioweapons have very strict requirements listed in Table 3.

**TABLE 3:** Summary of ideal Biological Warfare Requirements adapted and modified from Meyer and Morse (2008) and Christian (2013).

	**Requirements**	**Risk**	**Condition**
1	Availability in the Environment	Medium	Wide range of Rodent Host Reservoirs in North and south America
2	Ease of Design and Production	Medium	Possible Attenuation through Passaging Few Nonhuman Primate Lethal Disease Models Difficulty in Isolation and Purification of Virions New Technology improving yields and Virulence of Passaged Virions
3	Stability after Production and Persistence in the Environment	Medium	Long Durability and Persistence in Contained and Isolated Environments Sensitivity to Light and Heat
4	Effective Transmission Pattern and Routes of Entry	Medium	Inhalation of both ANDV and SNV Person-to-Person Transmission of ANDV
5	Effective Delivery Systems and Mode of Transportation	Medium	Effective Deployment Indoors Rodent Delivery is Onerous and Resource Intensive
6	Susceptible Target Population	High	Novel Emerging Infectious Disease with no known natural immunity within Human Populations
7	Absence of Specific and Effective Treatment including countermeasures that have the Ability of a Vaccine to Protect Certain Groups	High	No US FDA approved Antivirals or vaccines Some Antivirals and Vaccines for Old-World Hantaviruses with varying Degrees of Effectiveness
8	The Ability to Incapacitate or Kill Target Host	Medium	High Mortality Rate Low Morbidity Rate
9	Appropriate Particle Size for Aerosolization and Airborne Transmission	Medium	Can be Aerosolized Airborne Efficiency within Closed Environments
10	The Ability to be Disseminated in Food or Water Supplies	High	Can Contaminate Food and Water as well as commercial products
11	Logistic Requirements to Manufacture and Disperse Bioagents which include Infrastructural and Financial Support, Expertise, and Organizational Capabilities	Low	Intensive Laboratory Equipment and High Expertise Requirements High Costs Deployment of Infected Rodents Reservoirs is Demanding

*Table also includes the capabilities required by conventional or unconventional militaries to conduct and deliver a bioagent attack. Note that the ranking is unimportant. We also describe the risks associated with bioweapons requirements as they pertain to the feasibility of developing and deploying New-World Hantaviruses. Risk is graduated with regard to Low, Medium, and High.*

### HCPS-Causing Reservoirs Are Available and Are Affected by Environmental Factors

The presence of SNV-infected deer mice across the American Midwest is fairly high as seroprevalence of SNV antibodies were discovered in 38% of captured rodents in Indiana, with up to 25% of seroprevalence in the western US and 7% in the eastern US (Berl et al., 2018). SNV-infected deer mice are somewhat discontinuous across Canada, but are located in every Canadian province as well as the Yukon territory and tend to display greater than 30% seroprevalence in large, close proximity populations (Drebot et al., 2015). Seropositivity of ANDV was prevalent across South America, particularly Patagonia in Chile and Argentina with antibodies being present at 5.9% specifically for *Oligoryzomys longicaudatus* (Medina et al., 2009; Astorga et al., 2018). Male deer mice have a higher seroprevalence of SNV antibodies compared to female deer mice which is the same for *Sigmodontinae* species infected with ANDV (Padula et al., 2004; Medina et al., 2009). Consequently, acquiring HCPS-causing Hantaviruses is relatively easy and requires access to natural habitats and peridomestic environments that harbor the rodent reservoir. The relative abundance of HCPS-causing rodents will be dependent on precipitation but overall maintain high ecological densities (Jonsson et al., 2010).

Climate change will also have impacts to the acquisition and maintenance of Hantaviral reservoirs. Rodent population dynamics are particularly affected by a combination of unusually high rainfall followed by drought which is evidenced by the 1993 US Four Corners outbreak which was preceded by a dramatic increase in rainfall following the 1992–1993 El Niño warming phase (Gubler et al., 2001). These favorable conditions led to increases in rodent food sources and a significant increase in rodent population which took advantage of the Four Corners’ environment which provided favorable habitats conducive for the growth of *P. maniculatus* (Engelthaler et al., 1999). This likely contributed to rising deer mouse populations which resulted in increased exposure of rodent-human contact, similar to the PUUV outbreak in Northern Europe which was also precipitated by an unusually wet spring season which affected bank vole populations beneficially (Spiropoulou et al., 1994). The increase in North and Western European vole populations is adjusted by elevated average temperatures which improves mast production. Higher densities of rodents benefited from high seed production, itself improved by warmer summer conditions which benefited winter survival and subsequent spring breeding (Klempa, 2009). Incidentally, human-reservoir contact increased as the reservoir population increased.

Bioterrorist cells have the potential to take advantage of high rodent population densities. Having higher populations of asymptomatic but chronically infected rodent specimens can be utilized in either a one-target distribution model, or as a means to generate a critical concentration of passaged virions to achieve a weaponizable aerosol. Both methods would require a large-scale capture and maintenance of rodents, with the latter being more onerous in the process of passaging and isolating the hantavirus. However, the role of climate change provides access for bioterrorist groups to acquire the virus through freely available infected rodents because of their increased populations. This can change depending on the effects of human activity which is being accelerated by agricultural expansion, deforestation, land reclamation, irrigation projects, and infrastructural developments (Klempa, 2009).

### Difficulty in Cell Culturation Reduces Ease of Production

Hantaviruses have historically been very difficult to isolate and grow in both cell culture and animal models, which have limited their ability to be previously concentrated and weaponized (Chizhikov et al., 1995). The first successful passage of HTNV in a laboratory setting occurred in 1978, and the first successful passage and isolation of SNV occurred in 1994 (Elliott et al., 1994). The virus itself requires passaging by rodent-to-rodent transmission followed by cell culturing in Vero E6 cells, with the virus replicating specifically in *P. maniculatus* cells despite repeated attempts of using RT-PCR to amplify positive hantavirus from human or rodent samples (Elliott et al., 1994). Isolation from the reservoir host or from diseased human patients tends to require extensive blind passaging in cell culture to acquire adequate viral titres for characterization studies, with viral propagation being observed to elicit reduced infectivity in natural rodent reservoirs (Fulhorst et al., 1997; Galeno et al., 2002). SNV propagation in Vero cultures seems to cause mutations in the RdRp which potentially attenuates the virus and makes it less virulent (Safronetz et al., 2014). The problem arises from attempting to adapt the viruses to new hosts through sequential passaging from animal to animals as well as amplifying the virus in large stocks of Vero cell lines which have resulted in the attenuation of the viral culture (Prescott et al., 2017). Conversely, attempts at experimentally recreating signs and symptoms of HFRS or HCPS in a non-human primate model demonstrated that various non-human species can be infected by the disease but they do not develop obvious symptoms. This trend is observed in the attenuation of the Old-World Hantavirus PUUV in cell culture due to point mutations occurring in its S Segment. PUUV’s propagation in Vero E6 cells replicated with high efficiency but did not retroactively infect its natural reservoir host the bank vole (*Clethrionomys glareolus*) or cause severe disease in cynamalogous macaques (Lundkvist et al., 1997; Klingström et al., 2002; Eckerle et al., 2014). Comparatively, SNV propagated in deer mice after passaging in Vero cell lines elicited severe disease in its non-human primate model of rhesus macaques (Safronetz et al., 2014).

The previous reporting of hantavirus being difficult to isolate have also been attributed to the low concentrations of infectious virion particles extracted from the clinical or wild-caught infected rodents, with virion replication peaking at the time of death for the HCPS-infected human patients (Chizhikov et al., 1995). Combined with the slow and non-cytopathic growth of hantaviruses in cell culture are considerations as to why isolation becomes onerous (Chizhikov et al., 1995). Passaging has been successful in non-rodent, non-human primate models involving rhesus macaques, but they had to be previously passaged in deer mice to maintain virulence and infectivity which increases the requirements for weaponization (Safronetz et al., 2014; Warner et al., 2019a). Furthermore, SNV propagation in Vero cultures seems to cause mutations in the RdRp which potentially attenuates the virus and makes it less virulent (Safronetz et al., 2014). Consequently, the significant absence of any strong disease models outside of macaques and Syrian hamsters poses a challenge for weaponization, as the inability to replicate a similar human disease progression in primates from passaged and isolated virions will hinder the lethality of any engneered bioweapon.

Although tough and resource demanding, concentrating hantaviruses is not impossible and may become more efficient with newer technologies and techniques as Warner et al. (2019a) demonstrated. A way to increase viral stocks is to avoid using the standard intramuscular model of infection and instead use the intraperitoneal infection of deer mice which was demonstrated to produce SNV stocks with high viral RNA copy number (Warner et al., 2019a). New immunotherapies methods leading toward lethal disease models are also helping to increase the viral load as the infection of immunocompetent Syrian hamsters with cell-cultured SNV resulted in lower levels of viral dissemination compared to immunocompromised hamsters (Brocato et al., 2014; Vergote et al., 2017). Improvements in viral isolation for biological characterization studies has been conducted with HTNV and PUUV in suckling mice and Syrian hamsters respectively because of their sensitivity to infection (Seto et al., 2011; Li et al., 2013). There is still a reliance on Vero cell lines for viral propagation which has its own challenges. Vero E6 cells have been shown to produce an IFN-λ response to Hantaviral infection consequently reducing viral yields and affecting their quality (Prescott et al., 2010). The challenge of viral isolation, culturing, and modification in recent years has become relatively easy as indicated by the isolation and sequencing of the SARS-CoV-2 virions which demonstrates that synthetic biology methods are available for facilitating virion production which could include reverse engineering (Thao et al., 2020). Hantavirus components and virions as well as pseudovirions are already produced by passaging in Vero E6 cell lines, with RT-PCR methods and Vesicular Stomatitis Virus vectors being employed for sequencing and for the detection of hantavirus infection through the presence of their neutralizing antibodies (Elliott et al., 1994; Higa et al., 2012; Niskanen et al., 2019).

Additionally, given the rise in genetic engineering tools and techniques such as TALEN and CRISPR/Cas9, the ability to synthetically engineer more pathogenic bioagents is available (Benjamin et al., 2016). CRISPR/Cas9 was employed to reduce HIV viral replication in infected T-cells, and could potentially be employed to increase virulence and viral replication for other pathogens including HCPS-causing hantaviruses (Ophinni et al., 2018). Incidentally, the limitation of culturing hantaviruses virions now may be improved overtime with developments in gene editing and Do-It-Yourself technologies which have made sophisticated techniques more accessible to conventional militaries and terrorist organizations (Quetier, 2016).

### Susceptible Targets

#### Conventional Warfare Settings

Militaries and hantaviruses have a deep history which is largely tied with the operation of war and the requirements that are needed to support it (Johnson, 2001). One important factor of militaries is their strength component, comprised of large bodies of soldiers congregating in theaters of operations for extended periods of time. This has the unfortunate consequence of consolidating resources, especially food that has the tendency to attract animals such as rodents and insects, as well as disrupting natural habitats that affect ecosystems and the reservoirs that inhabit them (Lawrence et al., 2015). Warfare also extends disruptions to manmade infrastructure which generally creates barriers to illnesses, including housing and sanitation and the access to medical care facilities that could prevent the spread of diseases. A variety of these factors could be taken advantage of in warfare, whether it be a passive allowance of weakening military strength in the face of soldiers’ worsening living conditions, or the intentional spread of a pathogenic biological agent by natural vectors or artificial delivery systems. Like other major wartime diseases like Influenza and Typhoid Fever, hantaviruses have been identified in a variety of different conflicts.

Puumala virus causes a milder form of HFRS called Nephropathia Epidemica (NE) and is spread by the bank vole in Europe (Vapalahti et al., 2003). It is suspected that hantaviruses, specifically PUUV, spread across Europe during WWI in the form of Trench Nephritis which can be attributed to the congestion of soldiers and rodents in tight places, including trench lines that destroyed farmland and undermined infrastructure (Johnson, 2001; Schmaljohn, 2009; Lameire, 2014). Trench diseases, including Trench Foot, Trench Fever, and Trench Nephritis, constituted 25% of the British Expeditionary Force’s triage bed occupancy, and when the US entered the war in 1917, 0.54% of their 370,000 military personnel were affected by NE (Lameire, 2014). HFRS and hantaviruses as a whole came to the attention of western medicine during the Korean War (1950–1953) which observed 3,200 United Nations troops becoming infected, with HTNV being isolated and identified as the etiological agent in 1978 (Gajdusek, 1962; Lee et al., 2004). Similarly, in the early 1930s, Soviet troops encountered a similar disease along the Amur River that caused nephritis, bleeding, and shock while Imperial Japanese forces suffered 12,000 cases as they invaded Manchuria during the Second Sino-Japanese War (1937–1945) (Schmaljohn, 2009; Lameire, 2014). Aside from the 1993 HCPS outbreak, major hantavirus outbreaks such as HTNV and PUUV are associated with war.

Consequently, the military is a natural target for hantavirus as a result of their activities occurring in largescale field exercises or in land-based combat which can disrupt natural habitats and cause exposure to hantaviruses by dispersing HCPS-causing rodents as was the case for HFRS-causing *Apodemus agrarius* or the striped field mouse (Clement et al., 1996). HCPS cases continue to be reported following military personnel encounters with the rodent reservoir, especially in large-scale military exercises that overlap with the rodent reservoirs’ habitats (Parkes et al., 2016). The congregation of soldiers in poorly ventilated or rarely maintained defenses such as trench lines or housing complexes are at risk to the infestation of HCPS-causing rodents regardless of SNV or ANDV’s weaponization. As defenses and facilities decay overtime due to resource scarcity and war attrition, the ability to maintain sanitation and regular hygiene will be compromised enabling a return of Trench Nephritis and HCPS pulmonary disease. This can be further exacerbated by the influx and settling of refugees in consolidated camps which lack proper infrastructure and sanitation to prevent the spread of diseases let alone maintain barriers to hantavirus reservoir spread. This is evident by the influx of refugees generated from the conflict during the Yugoslav Wars (1991–2001) where civil unrest and internecine conflict caused massive movements of people and resulted in military and civilian exposure to hantaviruses, including the novel, HFRS-causing hantavirus DOBV which inflicted a 20% mortality rate (Markotic et al., 1996; Bugert et al., 1999). Incidentally, as infrastructure decays or is undermined by war, more people will be exposed to debilitating hantaviruses as contact with rodent reservoirs increases.

Within the range of the military, it would be appropriate to develop strategies to delay or inundate military forces by exposure to hantavirus through natural infection models. This would observe HCPS-causing hantaviruses to be deployed as area denial weapons which are employed to slow the advance or endanger target militaries. Area denial weapons tend to restrict the momentum of target forces, usually forcing them into positions of vulnerability which may include adopting additional precautions to manage and mitigate the effects of the bioweapon itself. Employing New-World Hantaviruses in this respect, whether it be the physical dispersal of HCPS-infected rodents to undermine the entrenched living conditions of soldiers, or the deployment of aerosolized virion particles would significantly affect the morale, strength, and movement of the target army. A strategy of area denial would be to harbor HCPS-causing rodents in built-up areas to prevent the appropriation of urban infrastructure by an invading force. Abandoned facilities would be especially exceptional since SNV is found highly aerosolized in small, ≤1 μm particulate matter that is far-more easily disturbed to the breathing zone (1.5 m height) from walking rather than sweeping (Richardson et al., 2013). Soldiers seizing urban areas would be most vulnerable, especially during the spring and summer months where reservoir breeding and particulate aerosolization increases (Waltee et al., 2009). SNV may also persist in excreta for longer since sunlight and UV radiation are blocked from actively degrading the virions due to the protection vacant buildings provide (Douglass et al., 2006).

#### Unconventional Warfare Settings and Civilian Targets

Targets to a country’s civilian populace or economic and industrial sectors are important alternatives for bioterrorist organizations or low-parity nations that cannot compete with modern industrial militaries. As illustrated by the 2019 SARS-CoV-2 pandemic, infectious diseases have the effect of compromising the entire socio-economic systems of countries and will be a practical target for most bioterrorist organizations. A bioweapons’ attack will likely force the civilian populace to seek shelter or undergo rigorous quarantine measures which will affect the consumption of products from primary and secondary sectors of industry. The 2019 SARS-CoV-2 pandemic’s quarantining especially reduced demand for oil and petroleum products, manufacturing, and agriculture worldwide as isolated civilians were no longer able to consume and grow the economy at previous rates resulting in a decline in overall national GDP (Nicola et al., 2020). Furthermore, impediments to the social fabric caused by the pandemic resulted in an overall abated pattern of life that observed closures of schools, increased hospitalization and pressures on the medical systems, as well as an increase in government debt and expenditure to maintain the stability of their financial sectors (Nicola et al., 2020). HCPS-causing Hantaviral bioweapons could be deployed in this way to afflict damage to a nation’s industrial output or to invoke panic amongst a civilian populace which would affect a country’s ability to fight conflicts abroad or domestically.

Farmers are naturally affected by the presence of hantaviruses due to their outdoor activities and cultivation of farmland which overlap rodent reservoir habitats (Vapalahti et al., 1999). A possible biothreat scenario involving hantavirus would likely target agriculture centers by increasing the incidence of contact with HCPS-causing rodents preventing farmers from working or by forcing them to require additional and costly protective equipment which would create delays in production. This includes traditional farmers utilizing lumber as a fuel source as firewood handling could result in the close contamination from Hantaviral infected aerosols or dust (Van Loock et al., 1999). The risk from storage or lumber shelters will especially affect those in the lumber and forestry industries and can thus be a target for a slow delivery in addition to an either targeted or widescale dispersal of hantavirus bioweapon which would delay or harass industrial production.

Hantaviruses, because of their global nature, have the capacity to affect infrastructure especially shipping and trade. Given a major outbreak, major ports contaminated with aerosolized hantaviruses have the capacity to create delays in trade which will endanger the economy of a target country. HFRS-causing SEOV is found worldwide because of its ubiquitous rodent reservoir the brown rat (*Rattus norvegicus*) whose close relationship with humans and subsequent dissemination through global trade, human migrations, and settlement has enabled its transit and viability (Lin et al., 2012). The presence of the brown rat in most major urban centers and in key transport industries such as maritime and land shipping can create a vulnerability to trade if targeted by bioterrorist organizations. Having a simple rodent infestation can threaten food stores and given hantaviruses general durability in moderate temperatures and low-UV light environments such as storage containers will allow aerosolized hantaviruses to survive up to 2-weeks and create hazards for government or civilian responders. Selective pressures and challenging environments like highlands, deserts, and cities will likely prevent dispersal of natural reservoirs of hantavirus such as deer mice. However, in North America, similar species to deer mice such as *Peromyscus leucopus* or the white-footed mouse have occupied effective niches in cities on the east coast of Canada and the United States and have taken advantage of urban environment’s lack of predators and natural competitors, its warmer climate for mating, and its abundance of small forest fragments for habitation (Munshi-South and Richardson, 2017). Attempting to build a natural reservoir in the city would take generations of rodent colonies and would itself be unviable given time, resources, and current rodent controls and proofing.

Hantaviruses also pose a risk to food consumption as well if improperly stored (Risteska-Nejashmikj et al., 2019; Wang et al., 2020). Hantaviruses, with PUUV and ANDV being studied, are not easily digested by stomach acids and can survive long enough to be passed into the gastrointestinal tract. Despite the requirements for intragastric route infection being the least effective, the oral route of infection is plausible for PUUV (Witkowski et al., 2017). Contaminating food and water supplies with biological weapons generally produces fewer casualties compared to an airborne release, but may be a secondary consequence resulting from a primary release (White, 2002). This would be the effect of having a warehouse contaminated with Hantaviral aerosols which will contaminate food stocks contained in tin cans or boxed containers. The consequence is two-fold. The first involves the vast stores of merchandise and material needing to undergo rigorous decontamination or disposal to prevent subsequent human contact and illness which will affect economic output. The second, if successful, will cause an indirect aerosol route of transmission to humans which could be widespread due to the nature of modern supply chains and distribution.

Hantaviruses are only currently pathogenic to humans as their rodent reservoirs remain chronically infected and asymptomatic of the disease, however, they continue to be highly viremic as the natural host produces antibodies including neutralizing antibodies (Netski et al., 1999; Ermonval et al., 2016). HFRS-causing hantaviruses are found to infect a wide array of rodents and insectivore species including bats, as well as hantavirus antibodies being found in domestic animals such as cats, dogs, rabbits, and pigs (Zhang et al., 2010). The infection of domestic animals and livestock such as cows is a concern because it produces another route of transmission between animals to humans. PUUV was demonstrated to experimentally infect bovine aortic endothelial cells, however, it is unknown whether asymptomatic persistent infections exist in domestic animals (Muranyi et al., 2004). Consequently, the effect on animals is fairly low as HCPS-causing hantaviruses do not cause disease in animals which remain largely asymptomatic (Krüger et al., 2011). This includes monkeys, with the only non-human primate exception being SNV-infected rhesus macaques and ANDV in Syrian hamsters which experienced severe HCPS-disease as a result of Vero E6 propagated virions (Hooper et al., 2001; Safronetz et al., 2014). Incidentally, targets for livestock and agriculture within bioterrorism attacks are very unlikely when using hantaviruses.

One of the limitations of hantaviruses is that they spread by specific rodent host species with most being spread by one or a few closely related rodents which reflect the co-evolutionary relationships hantaviruses generally have with their reservoirs (Hughes and Friedman, 2000). Hantavirus evolution and reassortment is limited to intraspecies reassortment and inter-lineage events within the same, single rodent reservoir (Klempa, 2018). Like Influenza, Hantaviruses are segmented and are able to undergo reassortment events with the exchange of gene segments between viruses that infect the same cells. The formation of antigenic shifts through reassortment events act as new ways for segmented viruses to adapt to new animal hosts and to increase infectivity. This can result in the formation of novel progeny viruses that are genetically distinct from the parental viruses and could be employed as the method of developing more pathogenic hantaviruses by bioterrorist organizations, especially with different HCPS-causing reservoir hosts co-located in close proximity (Klempa, 2018).

ANDV and SNV are genetically distinct hantaviruses that circulate in different regions and different rodent reservoirs. Despite ANDV not being maintained in deer mice, it can infect the SNV rodent reservoir allowing for new serotypes to occur (Ermonval et al., 2016). Additionally, ANDV and SNV reassortment events produced diploid and monoploid viruses with SNV S and L Segments and ANDV M Segments, which efficiently replicated in Vero E6 cells (Rizvanov et al., 2004). Infectivity of these new viruses takes on the characteristics of the ANDV M Segment they have adopted, and is suggested that reassortments of M Segment substitutions promote virus survival by increasing its infectivity (Rizvanov et al., 2004). Previous *in vitro* studies have observed the mixing of distinct strains of SNV in Vero E6 cells generating new reassorted viruses (Rodriguez et al., 1998). Pathogenic SNV NMR11 strains were also able to reassort with non-pathogenic Black Creek Canal Virus (BCCV), a distantly related New-World Hantavirus that infects a different rodent species, the cotton rat (*Sigmodon hispidus*) (Rodriguez et al., 1998). Despite the low frequency of reassortment and the lack of predominance of any specific segment over the other, the ability for the strains to reassort highlights the importance reassortment as a genetic mechanism is in the emergence of new and possibly more lethal hantaviruses (Rodriguez et al., 1998).

Naturally occurring SNV reassortments are rather limited though and generally occur within local deer mouse populations just because of the local ecology supporting so few rodent species who rarely encounter each other and allow natural reassortment to occur. This is also undermined by the increasing genetic distance between rodent species that make reassortments less frequent (Henderson et al., 1995). However, bioterrorist organizations can artificially force these interactions by ensuring infected rodent species are grouped together with similar species like bats, voles, or shrews to enable the reassortment of pathogenic hantaviruses to form. This is similar to the case of a lethal genotype of ANDV, *Araraquara orthohantavirus* (ARQV) being documented in neotropical bats in Brazil which exposes the possibility of creating recombinant viruses with more infectious and morbid segmented negative-sense RNA pathogens such as Ebola Virus or Influenza-type viruses (Sabino-Santos et al., 2018). The threat of reassortment enables hantaviruses to develop new opportunities to host-switch. This is especially important since mixing M Segments and their expressed glycoproteins enable the virus to interact with cell membrane proteins for entry, creating new routes of entry and new cell targets from old viruses (Klempa, 2018). Considering that reassortments can be done *in vitro*, the opportunity for bioengineering by random reassortments of pathogenic and non-pathogenic hantaviruses is possible.

### Transmission

Compared to other hantaviruses like PUUV which readily transmits between bank voles and persists effectively within the environment, SNV retains some limitations to its transmission both horizontally between rodents and vertically to humans (Kallio et al., 2006). SNV horizontal transmission between its rodent reservoir has been observed to occur through biting and scratching, frequently among males with indirect transmission being possible among laboratory-inoculated rodents (Bagamian et al., 2012). Although transmission from contaminated excreta is possible, freshly infected deer mice were more likely to shed the virus and transmit it horizontally at the 14 days post-infection stage where SNV replication appeared the highest (Warner et al., 2019a). These recent studies of horizontal infection between deer mice discovered that SNV-infection only occurred in 24% of the uninfected deer mice caged with a same-sex SNV-infected mate for 6 weeks. Additionally, subsequent experiments accounting for long-term shedding noticed no further uninfected deer mice contracting SNV from uncleaned cages alone (Warner et al., 2019a). In contrast, ANDV transmitted more efficiently between uninfected cage mates whilst maintaining higher persistence in the environment (Padula et al., 2004). Additionally, reproductively active males with wounds comprised the majority of ANDV seropositive *Oligoryzomys longicaudatus* rodent members with horizontal transmission being primarily through male intersexual competition (Juan et al., 2019). This offers a way to increase transmission horizontally to amplify hantavirus presence in the environment but is itself a difficult and resource consuming method. Ultimately, the positive pressure of SNV infection horizontally is limited to direct and aggressive interactions within the reservoir which affects how quickly a reservoir can be infected and dispersed against a military target. This also affects the cultivation of Hantaviral virions for concentration as the viral replication is impeded by the slow infection rate between deer mice.

While all hantaviruses are spread to humans via the inhalation of contaminated dust and aerosols dispersed from rodent feces, urine, saliva, and fur, the viability of its spread is limited to peridomestic risk areas such as barns, cabins, or warehouses (Douglass et al., 2006; Lonner et al., 2008). The extent of SNV infection is thus restricted to the presence of deer mice as the main delivery system until the foundation of more effective passaging and isolation techniques arise to make artificial airborne dispersal techniques more effective.

Unlike SNV and other hantaviruses, ANDV has a distinguishable route of transmission because of its ability to spread person-to-person exemplified by several small cluster outbreaks in Southern Chile and Argentina (Toro et al., 1998; Martinez et al., 2005). For person-to-person transmission to occur, close contact is required which increases the risk to people living in the same household as well as sexual partners. The presence of ANDV in the alveolar epithelium and salivary glands of *Sigmondontine* rodents reinforces intraspecies transmission from saliva and biting (Padula et al., 2004). ANDV infected patients have shown the virus to be present in pneumocytes and pulmonary macrophages, with ultrastructural and immunocytochemical studies revealing viral replication occurring in the alveolar epithelial cells with virus-like particles being released into the alveolar lumen (Pizarro et al., 2019). ANDV is likely secreted into human saliva and transmitted through close, intimate encounters or by exposure to respiratory droplets released through coughing or sneezing. There has also been reports of person-to-person transmission of ANDV from breast milk to new-borns, compounded by the new-borns’ inadequate immune system and the presence of vRNA in the breast milk (Bellomo et al., 2020). Nevertheless, ANDV person-to-person transmission appears to be limited to close contacts and not nearly similar to the transmission rate and basic reproduction numbers (R_O_) of SARS-CoV-2 or Category A Pathogens such as Ebola Virus which have been assessed to be greater than 1 (Althaus, 2014; Park, 2020). ANDV’s R_O_ number has been estimated to be significantly less than 1 and would likely not initiate a pandemic within the parameters of the current data (Woolhouse et al., 2016). Incidentally, the risk, albeit present, is rather limited because the efficacy of ANDV being rapidly disseminated throughout a target group is dependent largely on aerosol inhalation or contact with contaminated saliva with the latter being an unpractical method to strike at large target populations.

### Dispersal and Delivery

Since hantaviruses are transmitted to humans from rodents, a rudimentary but deliberate release of infected rodents into a target location would be a relatively easy way to threaten public health (Lõhmus et al., 2013). The impact would be low, but a strike against a country’s key infrastructure like trade ports, warehouses, hospitals, governments centers, or public gatherings with infected rodents would create delays to productivity and the economy. Modern, industrialized countries in the west would not undergo famine or experience food insecurity as a result of a biological attack to the agriculture sector because of its robustness in diversity, high-production and heightened regulation (Wheelis et al., 2009). However, disruptions caused by the presence of suspected or confirmed biological agents has the potential to inflict market speculation and contraction through bans on international food exports resulting in lost revenue, job losses, and the destruction of capital including livestock or contaminated merchandise. This is indicated by the pig and cow culling during the Foot and Mouth Disease and the Bovine Spongiform Encephalopathy outbreaks in the United Kingdom, United States, and the Republic of China in 1997 and the early 2000s (Dudley and Woodford, 2002). The intent would be to cripple infrastructure and overburden the economy and medical apparatus. If introduced into a target rural or urban area, infected rodents have the potential to cause long-term medical incidents and create public panic that will have the effect of consuming municipal or federal resources required to manage the attack (Lõhmus et al., 2013). Additionally, retaining and cultivating deer mice for Hantaviral preparation is cost effective and commercially available. Deer mice are easily maintained following standard laboratory mouse protocols with deer mice being no different from lab mice in terms of handling with the exceptions of their aggressive tendencies involving biting and their agility resulting in escape which prompts increased biosafety measures to be taken (Joyner et al., 1998; Martin et al., 2016). Deer mice also suffer from not being genetically homogeneous resulting in inconsistency in experimentation due to widespread and significant genetic polymorphisms.

Rodent dispersal is discreet and innocuous, and can go unnoticed compared to bioweapons deployed by artillery, missiles, or by aerial deployment by aerosols. One limitation of Hantaviral deployment by artillery or missile is due to its 60°C heat sensitivity as any incendiary or kinetic deployment system would inactivate and degrade Hantaviral virions (White, 2002). The preferable deployment mechanism would be an aerosolization or powdered pathway which is undetectable and can achieve rapid dispersal over a wide area (White, 2002). Hantavirus delivery could benefit from similar dispersal methods employed to transport Anthrax or Ricin toxins such as letters and mailed packages due to their persistence in UV-free environments (Bhalla and Warheit, 2004). This becomes a problem since hantaviruses are undetectable in these delivery systems which urges for the development of new diagnostic and detection equipment. Hantavirus infections in humans are diagnosed with tedious enzyme-linked immunosorbent assay (ELISA), or IgM-capture tests to detect IgM antibodies and also RT-PCR detection of viral RNA in rodent or insectivore hosts (Vaheri et al., 2008). However, the limitation of hantavirus isolation would prevent it from being assembled into an effective aerosol which would require high Hantaviral concentrations. This would require substantial laboratory resources and technical expertise to maintain sufficient viral stocks for weaponization, and would prove to be the leading difficulty for uninitiated bioterrorist organizations in accomplishing. Additionally, aerosols dispersed outside during the day have the risk of being degraded by the viricidal properties of UV radiation which poses another limitation to outdoor dispersal (Kraus et al., 2005). The optimal route would be to have dispersal mechanisms deploy indoors to prevent the seizure of facilities by militaries or to create disruptions for civilian personnel employed in key industrial sectors.

There does exist substantial methods of rodent and pest controls that target deer mice through bait and trappings, structural proofing and rodenticides which have proven both economical and effective in preventing rodent entry to structures including underdeveloped residences (Glass et al., 1997; Hopkins et al., 2002; Baldwin et al., 2014). By culling or isolating rodents through said techniques, rodent controls help to minimize human-rodent contact and ultimately transmission. This is exemplified by other rodent-borne *Bunyaviruses* such as the Arenavirus Lassa Virus which experienced a reduction in seroprevalence proportional to reductions in its North-West African reservoirs (*Mastomys natalensis*, *Mastomys erythroleucus*, *Hylomyscus pamfi*) through the use of rodenticides and urban proofing that targeted rodent and human food stocks and housing (Mari Saez et al., 2018). However, complete seroprevalence reduction of Lassa Virus relied upon an 80% reduction in rodent population densities indoors and in peridomestic environments to avoid lateral viral transmission which becomes labor and resource intensive and may not be feasible in developing countries or those affected by war (Mariën et al., 2019). Nevertheless, deployment of rodent hosts as physical carriers of a hantavirus bioweapon would be seriously hampered by a proactive application of bait poisons, fumigant poisons, or non-poisonous measures including traps. However, handling of caught rodents through traps including diseased rodents is both labor intensive and increases the risks associated with hantavirus exposure (Meerburg et al., 2008).

ANDV virus would be the preferred model for dispersal if conducting a specific one-target attack with collateral to personnel within an immediate vicinity. This is because ANDV can occur within household person-to-person contact and can cause up to 25–35% mortality rates (Krüger et al., 2011). An attack on a single target with the intent of causing panic and successive but limited infections within a household would be an unideal although possible diversionary method for assassination. Household contacts of ANDV are at risk of developing HCPS infections within 4 weeks with ANDV vRNA being routinely detected in blood cells for up to 2 weeks before symptoms or anti-hantavirus antibodies arise (Ferres et al., 2007). This enables a person-to-person model to be employed for targets that require discretion since infection can take effect weeks after the attempt has been made compared to overt assassination or sabotage attempts which risk immediate suspicion and association. This also enables the virus to be spread asymptomatically within an infected group, although an influenza-like pandemic seems unfeasible due to the strict requirements of ANDV infection relying on person-to-person contact being very close (Krüger et al., 2011).

## Conclusion

With the limitations present, HCPS-causing hantaviruses are generally restricted to the Category C definition largely because their spread and ability to be concentrated in the laboratory faces difficult barriers. The feasibility of developing HCPS-causing bioweapons comes from the few strengths hantaviruses possess which includes its high mortality rate. Hantaviruses can also be easily dispersed through aerosols if limited to indoor facilities or warehouses with no insolation and can effectively target personnel – especially the military – operating in close proximity to rodent reservoir habitats. HCPS-causing hantaviruses also benefit from being difficult to treat since no Old-World Hantavirus antivirals or vaccines have effective specificity against them. However, the morbidity rate of New-World Hantaviruses is very low, with ANDV being a potential but somewhat viable agent because of its person-to-person transmission pathway which would likely infect more people. Additionally, the ability to manufacture and produce ANDV or SNV into a lethal form that can be dispersed poses a problem to its weaponization due to the presence of attenuating mutations and absence of a strong disease model for non-human primates. Although widespread, the ability for HCPS-causing hantavirus reservoirs to adopt new urban environments is limited but may be improved by the effects of climate change and the increase in human industrial activity. As technologies improve and barriers to passaging and replicating hantavirus virions in culture become easier and more viable, the ability to mass-produce pathogenic HCPS-causing hantaviruses like SNV or ANDV may upgrade hantaviruses from a Category C to A definition.

Nevertheless, despite being a Category C pathogen, the threat of hantaviruses infections generally and the potential for it to be weaponized should not be ignored. Hantaviruses are emerging pathogens that require the attention of government and medical health research as globally they still occur frequently in developing countries with poor infrastructure or in rural, agrarian environments that have close contact with Hantaviral rodent-reservoirs. Hantavirus and HCPS continue to be a serious pathogen and disease to be considered carefully due to the environmental-associated risks of frequent rodent-human contact that expose military personnel, farmers and agriculture workers, and warehouse and shipping staff to the virus. The paucity of reporting in developing countries and the neglect that hantaviruses face allows it to slip under the radar and can be exploited by organizations that could potentially field extensive laboratory equipment and rodent reservoirs toward the development of hantavirus-based biological weapons. Infectious diseases generally can be mitigated with better reporting and surveillance, especially by monitoring the incidence of disease through extensive international health and medical networks. This can be accomplished by governments and academic agencies resolving to be proactive in testing, freely sharing clinical and experimental details, and maintaining intergovernmental transparency with regard to pandemics or the occurrence of bioweapon threats (Arthur et al., 2006). Naturally, with a stronger observation and tracking of infectious diseases the easier it is to identify and manage them when they occur.

Globally, the ability to employ biological agents is prohibited by the Biological and Toxin Weapon Convention (BTWC) ratified by the United Nations and 170 of their member states which has limited international biological warfare. However, because of their lack of inspection mechanisms, rogue states and terrorist organizations could circumvent the BTWC treaty and employ biological weapons against target nations (Jansen et al., 2014). The intent may not be to singly destroy a nation or completely kill its people, with terrorist objectives being more nuanced and complex such as the case with Al-Qaeda attempting to destabilize and disrupt US power in the Middle-East (Keeney and Von Winterfeldt, 2010). Instead, the importance of disruption is key since any bioterror attack regardless of its category could inflict damage to a nation’s populace, economy, and prestige which have deeper ramifications to global security. Consequently, further research into weaponization and surveillance are essential to prevent or mitigate the effects of bioweapons.

As a consideration, significant international and national cooperation must occur to safeguard global trade, public health, and international security from bioterrorism. Mitigation strategies against bioagent attacks can only be effective given the invested interests of governments and research scientists in protecting the health of their peoples. Incidentally, research into medical health science must be focused on building toward detection, identification, mitigation and management equipment and techniques with the concentration of resources from cooperating governments to fund developments in counter-terrorism and medical therapeutics. This would require an intergovernmental exchange of communication between research scientists, policy-makers, and the public to broaden transparency toward international security and scientific research (Zhao et al., 2020). Through cooperation, predictions of future attacks or employment of bioagents can be ascertained, preventing socio-economic collapses that could occur from industry-paralyzing infectious diseases.

## Author Contributions

MD’S and TP: conceptualization, formal analysis, writing, and visualization. TP: supervision and funding acquisition. Both authors read and agreed to the published version of the manuscript.

## Conflict of Interest

The authors declare that the research was conducted in the absence of any commercial or financial relationships that could be construed as a potential conflict of interest.
